# Acid‐Triggered Dual‐Functional Hydrogel Platform for Enhanced Bone Regeneration

**DOI:** 10.1002/advs.202415772

**Published:** 2025-01-27

**Authors:** Yao Xiao, Jinjin Ma, Xiaonan Yuan, Huan Wang, Fengyu Ma, Jun Wu, Qianglong Chen, Jie Hu, Lijie Wang, Zhendong Zhang, Chao Wang, Jiaying Li, Weishan Wang, Bin Li

**Affiliations:** ^1^ The First Affiliated Hospital of Shihezi University Shihezi Xinjiang 832000 China; ^2^ Medical 3D Printing Center Orthopedic Institute Department of Orthopedic Surgery The First Affiliated Hospital School of Basic Medical Sciences MOE Key Laboratory of Geriatric Diseases and Immunology Suzhou Medical College Soochow University Suzhou Jiangsu 215000 China; ^3^ Sanitation & Environment Technology Institute of Soochow University Suzhou Jiangsu 215163 China

**Keywords:** acidic microenvironment, Arg‐CDs metabolism, endogenous stem cells recruitment, osteogenesis and angiogenesis coupling

## Abstract

Stem cell implantation holds promise for enhancing bone repair, but risks of pathogen transmission and malignant cell transformation should not be ignored. Compared to stem cell implantation, recruitment of endogenous stem cells to injured sites is more critical for in situ bone regeneration. In this study, based on the acidic microenvironment of bone injury, an HG‐AA_1:1_‐SDF‐1α composite hydrogel with a dual‐control intelligent switch function is developed by incorporating stromal cell‐derived factor (SDF‐1α), arginine carbon dots (Arg‐CDs), and calcium ions (Ca^2+^) into the oxidized hyaluronic acid/gelatin methacryloyl (HG) hydrogel. The acidic microenvironment triggers the first switch (Schiff base bond is broken between HG‐AA_1:1_ and SDF‐1α) of HG‐AA_1:1_‐SDF‐1α composite hydrogel to continuously release SDF‐1α. Compared to the neutral (pH 7.4) media, the cumulative release of SDF‐1α in acidic (pH 5.5) media is ≈2.5 times higher, which enhances the migration and recruitment of endogenous mesenchymal stem cells (MSCs). The recruited MSCs immediately initiate the second switch and metabolize Arg‐CDs into the bioactive nitric oxide (NO) in the presence of Ca^2+^, activating NO/cyclic guanosine monophosphate (cGMP) signaling pathway to promote angiogenesis. Therefore, the engineered HG‐AA_1:1_‐SDF‐1α composite hydrogel shows promising potential to achieve “coupling osteogenesis and angiogenesis” for bone regeneration.

## Graphical abstract

In this study, HG‐AA_1:1_‐SDF‐1α composite hydrogel can respond to the acidic microenvironment to continuously release SDF‐1α, which recruited endogenous mesenchymal stem cells (MSCs) and immediately metabolize Arg‐CDs into the bioactive nitric oxide (NO) in the presence of Ca^2+^, activating NO/cyclic guanosine monophosphate (cGMP) signaling pathway to promote angiogenesis, showing promising potential to achieve “coupling osteogenesis and angiogenesis” for bone regeneration.

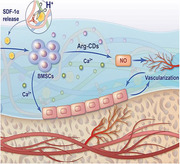

## 1 Introduction

During bone repair, mesenchymal stem cells (MSCs) can differentiate into osteoblasts, secrete various paracrine factors with immunomodulatory effects, and function in building regenerative microenvironments.^[^ ^1^ ^]^ Endogenous regenerative medicine is a promising new avenue in tissue engineering that focuses on mobilizing the homing of endogenous stem cells^[^ ^2^ ^]^ due to the body's own regenerative capacity to manage diseases. This approach not only enhances self‐repair in tissue, but also reduces the risks associated with cell transplantation, such as severe infections induced by pathogen transmission and malignant transformation.^[^ ^3^ ^]^ However, protection of the recruited endogenous MSCs from the hostile microenvironment in the target tissue often found at bone injury sites and further promoting osteogenic differentiation of the recruited MSCs to achieve effective bone regeneration remains an enormous challenge.^[^ ^4^ ^]^

Bone healing is a complex biological phenomenon and an intricate regenerative process including three successive overlapping phases, namely inflammation, repair, and remodeling.^[^ ^5^ ^]^ In the inflammatory phase, the blood supply is interrupted due to the disruption of vascular integrity following bone injury. Therefore, reduced blood supply and increased anaerobic metabolism cause hematoma, infections, and inflammatory responses. This subsequently results in lactic acid accumulation and formation of an acidic microenvironment at the bone injury sites.^[^ ^6^ ^]^ With the transition of the healing process into the repair phase, the pH at the bone injury site becomes a pivotal factor affecting various aspects of bone regeneration. Specifically, it significantly affects the process of bone regeneration, including migration, adhesion, proliferation, differentiation of osteoblasts, maturation of bone matrix secretion, inflammatory response, and angiogenesis.^[^ ^7^ ^]^ Therefore, generation of a more favorable pH environment may boost the survival and proliferation of MSCs, potentially enhancing the healing process.^[^ ^8^ ^]^

Suitable conditions for the growth of MSCs and efficient promotion of osteogenic differentiation of MSCs are key to bone regeneration.^[^ ^9^ ^]^ Bone defects are accompanied by vascular damage and bleeding, leading to a hypoxic state that induces inflammation and cell necrosis and hinders bone regeneration.^[^ ^10^ ^]^ Therefore, revascularization is a crucial factor in bone repair. This process involves the formation of new blood vessels, which is essential for delivering oxygen, nutrients, and growth factors to damaged bone tissue.^[^ ^11^ ^]^ Noteworthy, an adequate blood supply is vital for supporting regenerative processes and ensuring the survival of newly‐formed bone cells.^[^ ^10^, ^12^ ^]^ Nitric oxide (NO) is an important bioactive substance that mediates diverse signaling pathways in target cells and induces vasodilation during the inflammatory phase, thereby increasing the blood flow to fracture healing tissues.^[^ ^13^ ^]^ Moreover, NO activates the NO‐cyclic guanosine monophosphate (cGMP) signaling pathway and coordinates the osteogenic–angiogenic coupling effect to accelerate bone repair.^[^ ^14^ ^]^ L‐arginine (L‐Arg) is an important donor for in vivo NO production, a process catalyzed by NO oxide synthase (NOS).^[^ ^15^ ^]^ Endothelial NOS (eNOS), an NOS isoform, is a major regulator of vascular function. Activity of eNOS is regulated by increased intracellular calcium (Ca^2+^) concentrations, which triggers the production of NO in cells.^[^ ^16^ ^]^ Therefore, targeting the NO pathway, in particular, through the manipulation of L‐Arg metabolism and eNOS activity, could be a promising avenue for vascularization and improvement in osteogenesis of MSCs.^[^ ^17^ ^]^

**Figure 1:**
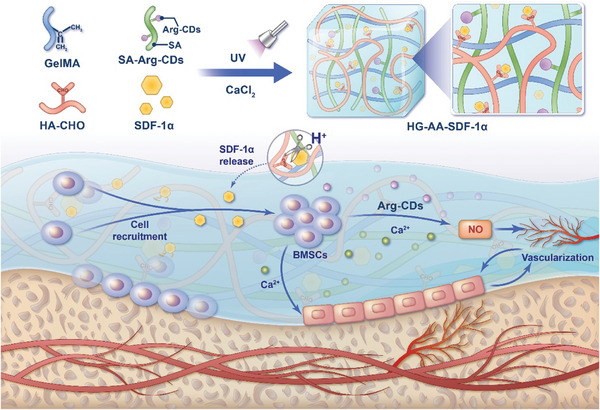
Schematic illustration of the HG‐AA‐SDF‐1α composite hydrogel enhances “coupling osteogenesis and angiogenesis” for the promotion of bone regeneration. HG‐AA‐SDF‐1α composite hydrogel releases SDF‐1α under the stimulation of the acidic microenvironment after bone injury. The released SDF‐1α rapidly recruits endogenous BMSCs to the bone injury area and immediately metabolize Arg‐CDs into the bioactive NO in the presence of Ca^2+^, exhibiting excellent angiogenesis. Moreover, the release of Ca^2+^ combined with the formation of blood vessels synergistically promoted bone regeneration.

In this study, to recruit and stimulate the osteogenic differentiation of MSCs in the bone damage area, a composite hydrogel with a dual‐control intelligent switch function was engineered (**Figure** **1**). Initially, the pre‐developed Arg carbon dots (Arg‐CDs) were combined with sodium alginate (SA) through the reaction of amino group on Arg‐CDs and carboxyl group on SA. Next, the mixture was combined with methacryloyl gelatin (GelMA), oxidized hyaluronic acid (HA‐CHO), and stromal cell‐derived factor‐1α (SDF‐1α). The HA/GelMA/SA‐Arg‐CDs/SDF‐1α (HG‐AA‐SDF‐1α) composite hydrogel was obtained after ultraviolet (UV) irradiation and Ca^2+^ coupling. The Schiff base bond formed between SDF‐1α and HA‐CHO in HG‐AA‐SDF‐1α could respond to and improve the acidic microenvironment at the bone injury sites, which intelligently releases SDF‐1α, thus rapidly recruiting bone marrow MSCs (BMSCs). The recruited BMSCs could metabolize Arg‐CDs to produce NO in the presence of Ca^2+^ released from HG‐AA‐SDF‐1α, which significantly promoted angiogenesis and provided a suitable osteogenic microenvironment for BMSCs at bone injury sites. Furthermore, free Ca^2+^ released from HG‐AA‐SDF‐1α showed excellent effects on the promotion of osteogenic differentiation of BMSCs. Consequently, HG‐AA‐SDF‐1α composite hydrogel engineered herein realizes “coupling osteogenesis and angiogenesis” and synergistically promotes bone regeneration under the stimulation of the acidic microenvironment of bone injury, and provides an excellent strategy for bone regeneration.

## 2 Results

### 2.1 Synthesis and Characterization of Composite Hydrogels

**Figure 2:**
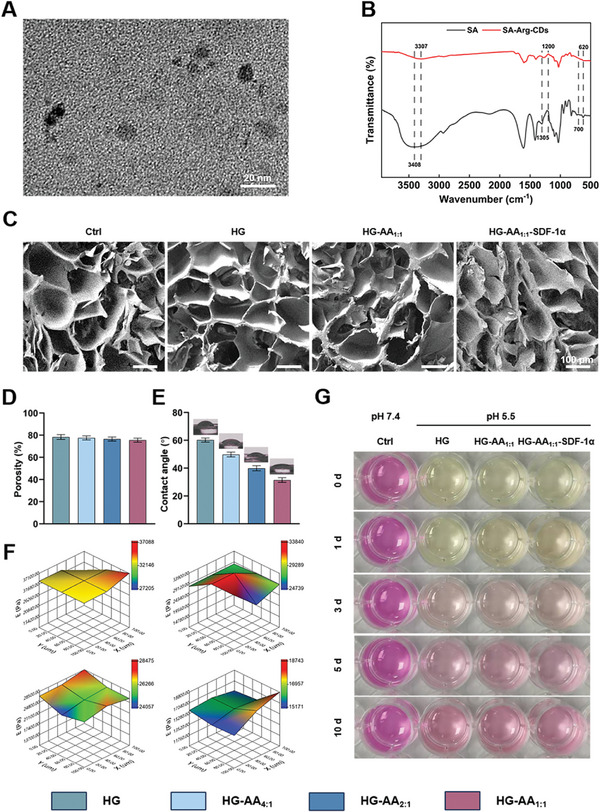
Characterizations of Arg‐CDs and composite hydrogels. A) TEM image of Arg‐CDs. B) FTIR of SA and SA‐Arg‐CDs. C) SEM images of composite hydrogels. D) Porosity of the composite hydrogels. E) Contact angles of the composite hydrogels. F) The 3D plot of nanoindentation tests. G) Color changes of media soaked in hydrogels. HG‐AA_4:1_, HG‐AA_2:1,_ and HG‐AA_1:1_ refer to the weight ratios of HG to AA as 4:1, 2:1, and 1:1, respectively.

The Arg‐CDs were spherical, the diameter was ∼6 nm (**Figure** **2**A). The result of X‐ray Diffraction (XRD) shows that there was a broad diffraction peak at 21° –25° of *2θ*, demonstrating the relatively low crystallinity of Arg‐CDs. However, there were sharp absorption peaks appeared at 31.7°, 45.4°, and 56.5° of *2θ*, which is consistent with the lattice stripe structure of Arg‐CDs (Figure S1A, Supporting Information). Moreover, the ultraviolet‐visible (UV‐vis) absorption spectra showed notable absorption peaks at the wavelength of 220 and 305 nm, indicating that Arg‐CDs were successfully obtained after calcination (Figure S1B, Supporting Information). Figure 2B shows the Fourier‐transform infrared (FTIR) spectra of SA and SA‐Arg‐CDs (AA). The spectrum of SA polysaccharides exhibits a broad hydroxyl absorption peak at 3408 cm^−1^. Comparatively, the spectrum of AA shows the red shifting of the peak at 3408 cm^−1^ for SA to 3307 cm^−1^. This is attributed to the fact that the addition of Arg‐CDs affected the O─H bonding environment, resulting in a slight decrease in the vibrational frequency, which reflects the effect of the interaction between Arg‐CDs and carboxylates. Most importantly, with the addition of Arg‐CDs, an amide bond was formed with SA. Therefore, some significant red shifts occurred at 1305–1200 cm^−1^, and 700–620 cm^−1^. It indicates the successful linkage between Arg‐CDs and SA. Figure S2A (Supporting Information) shows the appearance of a new peak at 1731 cm^−1^ for HA‐CHO, indicating the C═O stretching of HA‐CHO, demonstrating the successful fabrication of HA‐CHO. Moreover, SDF‐1α, an important chemokine for recruiting MSCs, was added to the composite hydrogel. Figure S2B (Supporting Information) illustrates that the doping of SDF‐1α into the composite hydrogel led to the formation of Schiff base bonds between SDF‐1α and the composite hydrogel. Thus, a significant redshift occurred between 3000–2800 cm^−1^ due to the bending and resonance vibrations of the aromatic C═N double bond, indicating the successful doping of SDF‐1α into the composite hydrogel.

The preparation of HG‐AA‐SDF‐1α composite hydrogels can be divided into the following steps. First, the AA and HA‐CHO/GelMA (HG) were prepared separately. Then, different ratios of AA, HG, and SDF‐1α were added, and eventually HG‐SDF‐1α, HG‐AA_4:1_‐SDF‐1α, HG‐AA_2:1_‐SDF‐1α, and HG‐AA_1:1_‐SDF‐1α composite hydrogels were obtained by crosslinking. Figure 2C shows that the composite hydrogels exhibited well‐organized and homogeneously connected macropores. Moreover, the porosities of all composite hydrogels were similar (Figure 2D). Therefore, the addition of AA or SDF‐1α showed little effect on the structure of the composite hydrogels. Figure 2E demonstrates that the contact angles of the other groups were smaller than that of the HG group, indicating the improvement in the hydrophilicity of the composite hydrogels after the addition of AA. HG‐AA_1:1_‐SDF‐1α was more hydrophilic and exhibited the smallest contact angle of 31.25 ± 2.25°, which was more suitable for cell attachment.

Subsequently, the swelling ratio and degradation of the composite hydrogels were evaluated. Figure S3A (Supporting Information) illustrates that all the composite hydrogels exhibited significant swelling properties after immersion in PBS solution. Moreover, the swelling ratio gradually decreased with the increase in the AA content, which was possibly attributed to the network structure of AA with a high cross‐linking density. Furthermore, the properties of in vitro degradation showed the same trends (Figure S3B, Supporting Information). The degradation rate gradually decreased with increasing AA concentration. These results demonstrate that the degradation properties of composite hydrogels could be finely tuned under different AA/HG ratios for subsequent in vivo applications.

Finally, Young's modulus of the composite hydrogels was evaluated by nanoindentation test. The results show that the modulus of HG‐SDF‐1α was 43.58 ± 5.44 kPa. After the addition of AA, the Young's modulus of HG‐AA_4:1_‐SDF‐1α, HG‐AA_2:1_‐SDF‐1α, and HG‐AA_1:1_‐SDF‐1α decreased to 38.27 ± 4.37, 26.34 ± 1.26, and 21.59 ± 1.58 kPa, respectively (Figure 2F). Despite the reduction in Young's modulus, all groups still showed satisfactory mechanical properties, which were conducive to maintaining structural integrity after implantation. Besides, the mechanical property of the composite hydrogels was measured. The results show that HG exhibited higher compressive strength than other groups. Moreover, the Young's modulus decreased with the addition of AA, and HG‐AA_1:1_ was about 20 ± 2.37 kPa (Figure S4A, B, Supporting Information). The rheological properties of hydrogels were also measured. The results show that the behavior of composite hydrogels was similar to that of elastic solids. As shown in Figure S5A (Supporting Information), the critical strain point for maintaining the hydrogel morphology was determined by strain amplitude scanning. We found that temperature and pH could affect the critical strain of the composite hydrogels. Taking 37 °C and pH 5.5 as an example, when the strain exceeded 215%, the loss modulus (G′′) exceeded the storage modulus (G′), indicating that the hydrogel was destroyed and transited to a quasi‐liquid state (Figure S5A, Supporting Information). In addition, the frequency scanning showed that the HG‐AA_1:1_ composite hydrogel exhibited a frequency independent behavior, that was, the storage modulus (G′) at different frequencies was higher than the loss modulus (G′′), thus the elastic network of HG‐AA_1:1_ composite hydrogel could be maintained when the frequency changed (Figure S5B, Supporting Information). Moreover, a remarkable feature of the HG‐AA_1:1_ composite hydrogel was its shear thinning behavior. At low shear rates, the viscosity of the composite hydrogel was high. However, with the increase of shear rate, the viscosity of composite hydrogels in different environments decreased. Moreover, the storage modulus of the composite hydrogel in the acidic environment decreased, indicating that the rigidity and elasticity of the composite hydrogel decreased, showing a softer behavior (Figure S5C, Supporting Information). Therefore, it could be better explained that the Schiff base bond in the composite hydrogel can break in response to the acidic environment and trigger the first switch of the subsequent cascade reaction.

After soaking the hydrogel in a medium at pH 5.5, the color of the medium gradually changed from yellow to red (Figure 2G). After 10 days of immersion, the pH of medium slowly increased to neutral levels. These results suggest that the hydrogels were able to improve the local acidic environment, which was favorable for cell survival, angiogenesis, and osteogenesis.

### 2.2 Characterization of Biocompatibility of Composite Hydrogels

**Figure 3:**
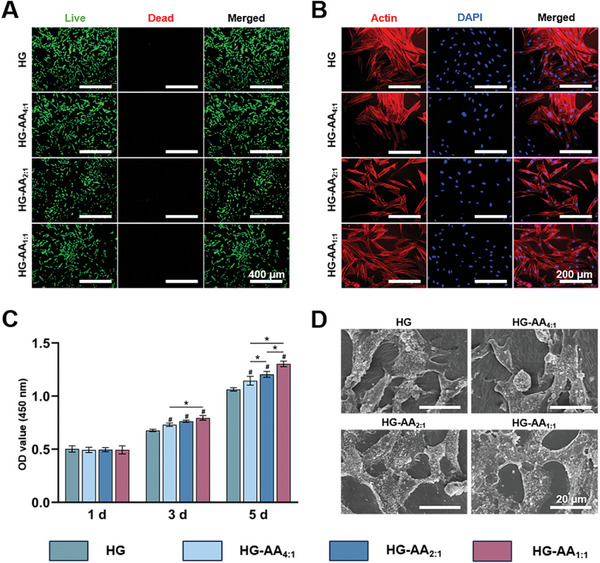
Cell morphology and proliferation on the surface of composite hydrogels. A) Representative Live/Dead staining images of BMSCs on composite hydrogels. B) Representative cytoskeleton staining images of BMSCs on composite hydrogels. C) Quantitative analysis of cell proliferation assay. Data are expressed as the mean ± SD (n = 6, ^#^ compared with HG group, ^*^ compared with each other; ^*^ *p* < 0.05) D) SEM images of cell morphology on composite hydrogels.

To further assess the biocompatibility, BMSCs and human umbilical vein endothelial cells (HUVECs) were seeded on composite hydrogels. The live/dead staining shows that there were almost no dead cells in all groups, indicating negligible cytotoxicity of the composite hydrogels (**Figure** **3**A; Figure S6, Supporting Information). Moreover, cytoskeleton staining showed that BMSCs and HUVECs adhered to and grew well on all composite hydrogels, exhibiting excellent biocompatibility (Figure 3B; Figure S7, Supporting Information). BMSCs and HUVECs spread well on composite hydrogels, and all cells on the composite hydrogels exhibited extended and flat filamentous pseudopods, clearly indicating that AA did not adversely affect the biocompatibility (Figure 3D; Figure S8, Supporting Information). Cell proliferation was assessed by cell counting kit‐8 (CCK‐8) (Figure 3C; Figure S9, Supporting Information). The results show that the number of cells increased continuously over a period of five days. Interestingly, with the addition of AA, the cell viability increased significantly, which was attributed to the enhancement by Arg‐CDs. To assess the long‐term stability of composite hydrogels, HG and HG‐AA_1:1_ were placed at 4 °C, −20 °C and −80 °C, respectively. After 12 h, the composite hydrogels were transferred to 37 °C for 2 h. Then BMSCs and HUVECs were seeded on the surface of HG and HG‐AA_1:1_. After 24 h, both HG and HG‐AA_1:1_ remained intact. Moreover, the live/dead staining shows that BMSCs and HUVECs adhered to and grew well on the surface of HG and HG‐AA_1:1_, maintaining excellent cell viability and demonstrating long‐term stability and biocompatibility (Figure S10, Supporting Information). Consequently, in view of its good biocompatibility and satisfactory promotion of proliferation, HG‐AA_1:1_ was used for subsequent experiments.

### 2.3 Evaluation of Cell Migration of Composite Hydrogels

**Figure 4:**
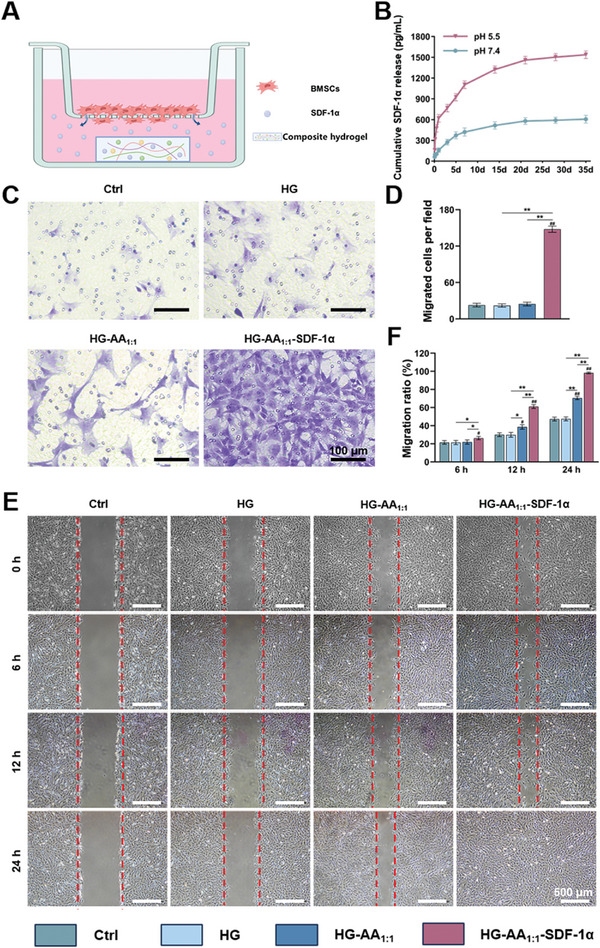
Chemotaxis of SDF‐1α released from HG‐AA_1:1_‐SDF‐1α. A) Schematic diagram of the BMSCs co‐culture model with composite hydrogels, created with biorender. B) Release curves of SDF‐1α from the composite hydrogels. C) Representative images of migrated BMSCs in the Transwell assay. D) Quantitative analysis of the migrated BMSCs in the Transwell assay (n = 8 images). Data are expressed as the mean ± SD (n = 8, ^#^ compared with HG group, ^*^ compared with each other; ^*^ *p* < 0.05, ^**^ *p* < 0.01) E) Cell scratch assay for BMSCs migration. F) Quantitative analysis of migration area (n = 6 images). Data are expressed as the mean ± SD (n = 6, ^#^ compared with HG group, ^*^ compared with each other; ^*^ *p* < 0.05, ^**^ *p* < 0.01).

To test the role of composite hydrogels in cell recruitment, BMSCs were seeded into the upper chamber of a 24‐well transwell plate, and the obtained HG‐AA_1:1_‐SDF‐1α composite hydrogels were placed into the lower chamber. **Figure** **4**A illustrates that the released SDF‐1α from the composite hydrogels could recruit BMSCs and promote cell migration. First, the release behavior of SDF‐1α from composite hydrogels was investigated. Next, to simulate the microenvironment of bone injury, SDF‐1α was measured in acidic (pH = 5.5) and neutral (pH = 7.4) media, respectively (Figure 4B). The cumulative release curve showed the sustainable delivery of SDF‐1α for at least 35 days. The release rate of SDF‐1α from HG‐AA_1:1_‐SDF‐1α was higher in acidic medium. After 35 days, the cumulative release of SDF‐1α in acidic (pH 5.5) media was ∼2.5 times higher than that in neutral (pH 7.4) media. It suggests that the Schiff base bonds were intelligently broken in acidic microenvironment, resulting in the release of SDF‐1α.

Further, a cell migration assay was performed to better evaluate the chemotactic effect of SDF‐1α on BMSCs. After 24 h, crystal violet staining showed the migration of more BMSCs to the lower chamber in the HG‐AA_1:1_‐SDF‐1α composite hydrogel group, indicating that the released SDF‐1α exhibited a significant cell recruitment effect (Figure 4C). Quantification of the number of migrated BMSCs showed significantly enhanced effect on cell migration when treated with HG‐AA_1:1_‐SDF‐1α composite hydrogel (Figure 4D). Furthermore, the scratch assay shows the healing rates of scratches in the HG‐AA_1:1_‐SDF‐1α composite hydrogel group reached ≈26.28%, 61.06%, and 99.23% after 6, 12, and 24 h, respectively, which were higher than those in the other groups (Figure 4E,F). Taken together, HG‐AA_1:1_‐SDF‐1α showed excellent cell recruitment effects due to the sustained release of SDF‐1α, thus showing enormous potential for recruiting cells to injured sites.

### 2.4 The Performance of Angiogenesis of Composite Hydrogels

**Figure 5:**
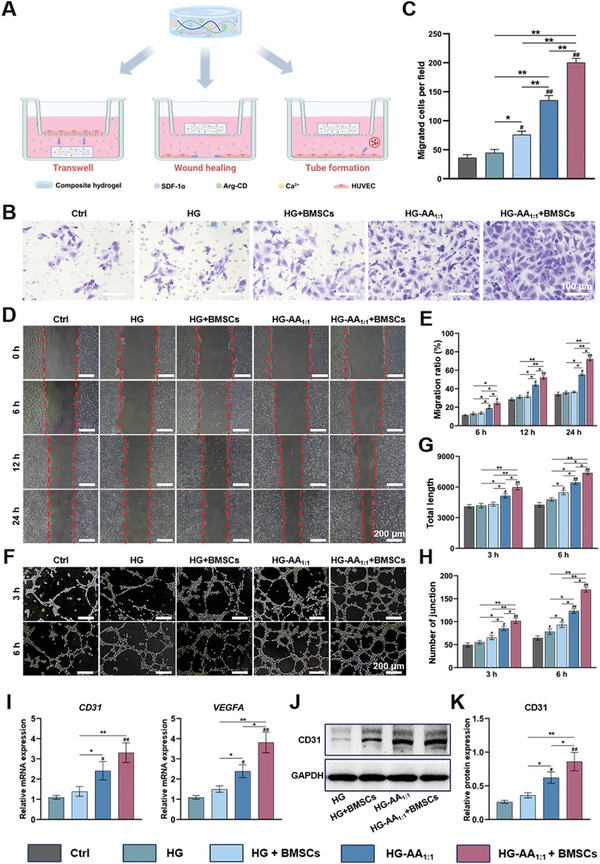
Stimulation of angiogenesis of HUVECs by the composite hydrogels in vitro. A) Schematic diagram of the co‐culture model of HUVECs and composite hydrogels, created with biorender. B) Representative images of migrated HUVECs in the Transwell assay. C) Quantitative analysis of the migrated HUVECs in the Transwell assay (n = 8 images). Data are expressed as the mean ± SD (n = 8, ^#^ compared with HG group, ^*^ compared with each other; ^*^ *p* < 0.05, ^**^ *p* < 0.01) D, E) Cell scratch assay and quantitative analysis of HUVECs migration area (n = 6 images). Data are expressed as the mean ± SD (n = 6, ^#^ compared with HG group, ^*^ compared with each other; ^*^ *p* < 0.05, ^**^ *p* < 0.01). F) Tube formation assay of HUVECs co‐cultured with composite hydrogels. G, H) Quantitative analysis of the total length and number of junctions formed by HUVECs in the tube formation assay (n = 5 images). Data are expressed as the mean ± SD (n = 5, ^#^ compared with HG group, ^*^ compared with each other; ^*^ *p* < 0.05, ^**^ *p* < 0.01) I) Relative quantification results of angiogenic genes in HUVECs cultured with the composite hydrogels. Data are expressed as the mean ± SD (n = 3, ^#^ compared with HG group, ^*^ compared with each other; ^*^ *p* < 0.05, ^**^ *p* < 0.01) J) The expression of angiogenic proteins in HUVECs cultured with the composite hydrogels. K) Relative quantification results of western blotting. Data are expressed as the mean ± SD (n = 3, ^#^ compared with HG group, ^*^ compared with each other; ^*^ *p* < 0.05, ^**^ *p* < 0.01).

In this study, HUVECs were co‐cultured with composite hydrogels to evaluate their angiogenic properties (**Figure** **5**A). Arg could be metabolized to produce NO via intracellular eNOS in the presence of Ca^2+^, thus endowing the composite hydrogel with excellent angiogenic properties. Therefore, BMSCs were seeded on composite hydrogels to promote NO metabolism, thus five groups (Ctrl, HG, HG+BMSCs, HG‐AA_1:1_, and HG‐AA_1:1_+BMSCs) were divided to assess the angiogenic properties. The results show the absence of significant difference in the migration of HUVECs after treatment with HG compared with the Ctrl group, whereas the number of migrated HUVECs increased in the HG‐AA_1:1_+BMSCs group (Figure 5B). Notably, HG‐AA_1:1_ contains several Arg‐CDs, thus the BMSCs on the surface of the HG‐AA_1:1_ composite hydrogel was able to catabolize Arg to produce NO for promoting angiogenesis. Compared with other groups, the number of migrated HUVECs in the HG‐AA_1:1_ and HG‐AA_1:1_+BMSCs groups increased significantly, almost five times more than that of the Ctrl group (Figure 5C). The result of the scratch test showed that the healing area in the HG‐AA_1:1_ group and the HG‐AA_1:1_+BMSCs group was larger than Ctrl and HG groups after 12 and 24 h, respectively (Figure 5D,E).

Tube formation of HUVECs is a key process during angiogenesis. Figure 5F demonstrates that compared with the Ctrl group, the number of tube structures in the HG‐AA_1:1_ and HG‐AA_1:1_+BMSCs groups increased significantly. Moreover, these two groups formed more mature and complete tubular structures than the other groups. Consistently, the quantification also showed that the tube length and number of branching points in the HG‐AA_1:1_ and HG‐AA_1:1_+BMSCs groups were higher than other groups (Figure 5G,H). In particular, HUVECs formed the longest tube and the highest number of branch points in the HG‐AA_1:1_+BMSCs group.

To further investigate the potential mechanism of composite hydrogels in angiogenesis, angiogenesis‐related genes and proteins were evaluated. Figure 5I illustrates that CD31 in HUVECs was upregulated after treatment with the HG‐AA_1:1_+BMSCs group compared with the other groups, further indicating that Arg‐CDs could be metabolized into NO by BMSCs in the presence of Ca^2+^ and showed excellent angiogenic properties. Moreover, the expression of vascular endothelial growth factor (VEGF) in HUVECs after co‐culturing with composite hydrogels was also assessed. Figure 5I exhibits that the HG‐AA_1:1_ and HG‐AA_1:1_+BMSCs groups showed significantly increased expression of *CD31* and *VEGFA* in HUVECs compared with the other groups. Moreover, western blotting was performed to confirm the effect of the composite hydrogel on angiogenesis, and significant enhancement in the expression of CD31 in HUVECs was also observed in HG‐AA_1:1_ and HG‐AA_1:1_+BMSCs groups (Figure 5J,K). Taken together, HG‐AA_1:1_ composite hydrogel enhanced the endothelial cell migration and formation of tubular structures, thereby exhibiting desirable angiogenic effects.

### 2.5 In Vitro Promotion of Osteogenesis of Composite Hydrogels

**Figure 6:**
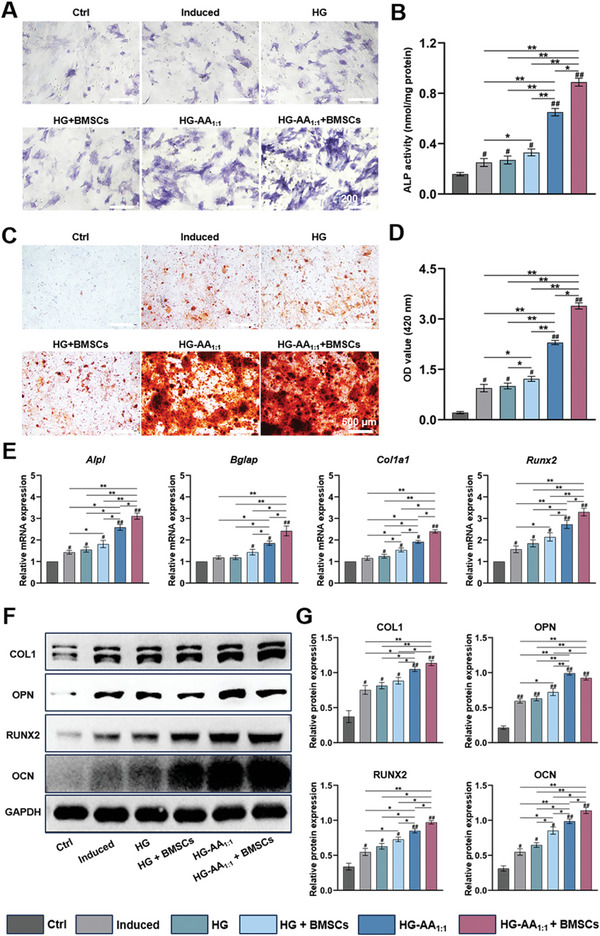
Stimulation of osteogenesis of BMSCs by the composite hydrogels in vitro. A) Representative images of ALP staining in BMSCs. B) Quantitative analysis of ALP activity in BMSCs (n = 5 images). Data are expressed as the mean ± SD (n = 5, ^#^ compared with HG group, ^*^ compared with each other; ^*^ *p* < 0.05, ^**^ *p* < 0.01) C, D) Representative images and quantitative analysis of calcium deposition of BMSCs (n = 5 images). Data are expressed as the mean ± SD (n = 5, ^#^ compared with HG group, ^*^ compared with each other; ^*^ *p* < 0.05, ^**^ *p* < 0.01). E) Relative quantification results of osteogenesis‐related genes in BMSCs cultured on the composite hydrogels. Data are expressed as the mean ± SD (n = 3, ^#^ compared with HG group, ^*^ compared with each other; ^*^ *p* < 0.05, ^**^ *p* < 0.01) F) The expression of osteogenesis‐related proteins in BMSCs cultured on the composite hydrogels. G) Relative quantification results of western blotting. Data are expressed as the mean ± SD (n = 3, ^#^ compared with HG group, ^*^ compared with each other; ^*^ *p* < 0.05, ^**^ *p* < 0.01).

To determine the promotion of osteogenesis of composite hydrogels, alkaline phosphatase (ALP) staining was carried out to assess the early effect of composite hydrogel on the in vitro osteogenic activity of BMSCs. **Figure** **6**A exhibits that HG‐AA_1:1_ and HG‐AA_1:1_+BMSCs composite hydrogels led to significant enhancement in the ALP activity in BMSCs compared with the other groups. Quantitative analysis further showed that the ALP activity in BMSCs cultured with HG‐AA_1:1_+BMSCs composite hydrogels was approximately five times higher than that of the Ctrl group, revealing optimal early osteogenic potential (Figure 6B). Mineralized nodules, a marker of osteogenic differentiation and maturation of BMSCs, were assessed by alizarin red staining. It was found that BMSCs cultured with HG‐AA_1:1_ composite hydrogel produced more calcified nodules, and the most calcified and denser nodules were produced in BMSCs treated with HG‐AA_1:1_+BMSCs composite hydrogels compared with the other groups (Figure 6C,D).

Osteogenesis‐related genes and proteins in BMSCs were evaluated by quantitative reverse transcription‐polymerase chain reaction (qRT‐PCR) and western blotting. The results revealed that HG‐AA_1:1_+BMSCs promoted the expression of osteogenic genes in BMSCs, such as alkaline phosphatase (*Alpl*), runt‐related transcription factor 2 (*Runx2*), collagen type I (*Col1a1*), and bone γ‐carboxyglutamic acid protein (*Bglap*) (Figure 6E). Similarly, the results of western blotting showed that the expression of osteogenesis‐related proteins, including RUNX2, osteopontin (OPN), type I collagen (COL1), and osteocalcin (OCN), was obviously higher in BMSCs cultured in HG‐AA_1:1_+BMSC group than those in the other groups (Figure 6F,G). These results further indicate that HG‐AA_1:1_+BMSCs could effectively promote osteogenic differentiation of BMSCs.

### 2.6 Mechanisms of Composite Hydrogels‐Mediated Modulation of Osteogenesis and Angiogenesis

**Figure 7:**
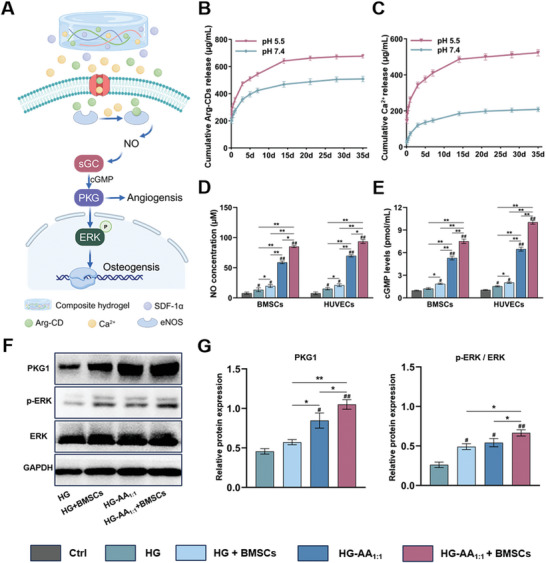
Effect of composite hydrogels on activation of the NO/cGMP signaling pathway. A) Schematic diagram showing that the released Ca^2+^ and Arg‐CDs synergistically activate the NO/cGMP pathway, ultimately promoting angiogenesis and osteogenesis, created with biorender. B, C) Release curves of Arg‐CDs and Ca^2+^ from the composite hydrogels under neutral and acidic conditions, respectively. D) Quantitative analysis of NO generation in BMSCs and HUVECs. Data are expressed as the mean ± SD (n = 5, ^#^ compared with HG group, ^*^ compared with each other; ^*^ *p* < 0.05, ^**^ *p* < 0.01). E) Quantitative analysis of cGMP expression in BMSCs and HUVECs. Data are expressed as the mean ± SD (n = 5, ^#^ compared with HG group, ^*^ compared with each other; ^*^ *p* < 0.05, ^**^ *p* < 0.01) F) Expression of PKG1 and ERK in BMSCs. G) Relative quantification results of western blotting. Data are expressed as the mean ± SD (n = 3, ^#^ compared with HG group, ^*^ compared with each other; ^*^ *p* < 0.05, ^**^ *p* < 0.01).

To investigate the potential mechanisms underlying osteogenesis and angiogenesis of composite hydrogels, generation of NO and activation of NO/cGMP signaling pathway were evaluated. In this study, the HG‐AA_1:1_‐SDF‐1α composite hydrogel responded to an acidic microenvironment to release SDF‐1α and recruit numerous BMSCs. Moreover, the released Ca^2+^ activated eNOS in BMSCs to catalyze the metabolism of Arg‐CDs to promote NO production. Finally, elevated NO levels further triggered downstream cascades that synergistically improved osteogenesis and angiogenesis (**Figure** **7**A). Herein, the release kinetics of Arg‐CDs and Ca^2+^ were assessed. The results show that the HG‐AA_1:1_‐SDF‐1α composite hydrogels consistently released Arg‐CDs and Ca^2+^, and the cumulative release of Arg‐CDs and Ca^2+^ in the acidic microenvironment was higher than that under neutral conditions (Figure 7B,C). Next, this study investigated whether a sustained adequate supply of Arg‐CDs and Ca^2+^ could synergistically promote NO production and activate downstream signaling pathways. First, the amount of NO produced by BMSCs and HUVECs was measured. Figure 7D exhibits that cell cultured with HG‐AA_1:1_ and HG‐AA_1:1_+BMSCs produced more NO, suggesting that Ca^2+^ released from the composite hydrogel promoted NO production by activating eNOS. Furthermore, the release of Arg‐CDs also enhanced the production of NO in BMSCs and HUVECs. Next, the expression of cGMP was measured by enzyme‐linked immunosorbent assay (ELISA) kits, and the cGMP concentrations in BMSCs and HUVECs cultured with HG‐AA_1:1_ and HG‐AA_1:1_+BMSCs were higher than other groups, demonstrating the activation of the NO/cGMP signaling pathway (Figure 7E). Moreover, the expression of the downstream signaling molecules including PKG1 and p‐ERK in the NO/cGMP pathway was also evaluated by western blotting, which further showed that HG‐AA_1:1_+BMSCs could promote the expression of PKG1 and p‐ERK. This result indicates that Arg‐CDs and Ca^2+^ released from the composite hydrogels could synergistically activate the NO/cGMP pathway (Figure 7F,G).

### 2.7 In Vivo Bone Repair Assessment

**Figure 8:**
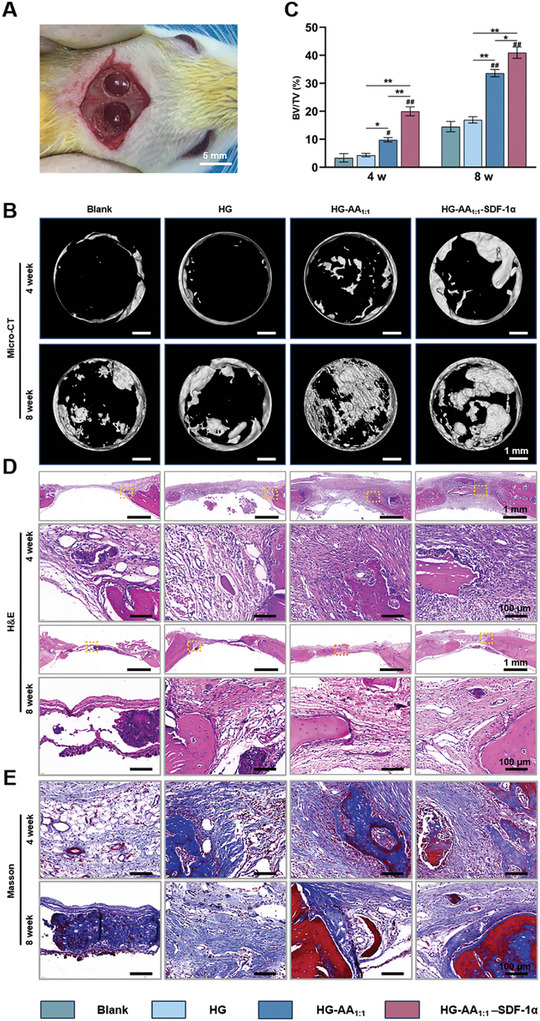
In vivo evaluation of bone regeneration repaired using composite hydrogels in a rat skull defect model. A) A critical‐sized skull defect model was created for bone regeneration evaluation of the composite hydrogels. B) 3D reconstruction of the defect areas at 4 and 8 weeks. C) Quantitative analysis of BV/TV in defect area. Data are expressed as the mean ± SD (n = 3, ^#^ compared with HG group, ^*^ compared with each other; ^*^ *p* < 0.05, ^**^ *p* < 0.01) D) H&E and E) Masson staining of defect areas repaired by composite hydrogels at 4 and 8 weeks after implantation.

**Figure 9:**
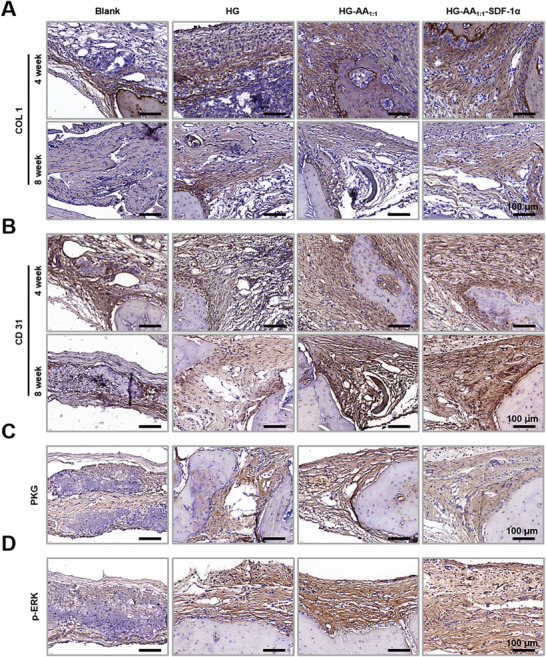
Evaluation of activation of NO‐cGMP signaling pathway of composite hydrogels in vivo. A, B) Immunohistochemical staining of (A) COLI, (B) CD31, C) PKG and D) p‐ERK in defect areas repaired by composite hydrogels.

SD rat cranial defect model was constructed to further evaluate the in vivo bone formation of HG‐AA_1:1_‐SDF‐1α composite hydrogels (**Figure** **8**A). Briefly, bone defects were drilled on rat skull, and HG, HG‐AA_1:1_, and HG‐AA_1:1_‐SDF‐1α were implanted into the defect area. Next, bone samples were collected and characterized at 4 and 8 weeks post‐operation, respectively. After 4 weeks, micro‐computed tomography (micro‐CT) results showed that the Ctrl group produced minimal new bone within defects, and the HG group exhibited almost equal amounts of bone formed at the defect site of the Ctrl group. Further, the HG‐AA_1:1_ group showed scattered island bone formation at the defects, but it could only cover a small portion of the defects, whereas the HG‐AA_1:1_‐SDF‐1α group formed new bone tissue covering the entire defect sites. After 8 weeks, almost the whole defect was repaired by HG‐AA_1:1_‐SDF‐1α (Figure 8B). Quantitative analysis of the new bone volume/tissue volume (BV/TV) ratio of newly‐formed bone tissue also confirmed this trend (Figure 8C). BV/TV ratio measurements showed that the amount of new bone in the HG‐AA_1:1_‐SDF‐1α group was 2–3 times higher than other groups. The results of hematoxylin–eosin (H&E) and Masson trichrome (Masson) staining were consistent with those of the micro‐CT analysis (Figure 8D,E). Moreover, to confirm the recruitment of BMSCs into the bone injured sites, immunofluorescence was performed 8 weeks after surgery. The result shows that a large number of CD105‐positive stem cells appeared at the bone injury site in HG‐AA_1:1_‐SDF‐1α composite hydrogel group compared to other groups, demonstrating HG‐AA_1:1_‐SDF‐1α composite hydrogel could significantly recruit MSCs from the surrounding area to enter the bone injury site and thus accelerate bone regeneration (Figure S11, Supporting Information). To further validate the osteogenic and angiogenic coupling of the composite hydrogels, immunohistochemical staining was carried out for COL1 and CD31 as osteogenic and angiogenic markers, respectively. Clearly, HG‐AA_1:1_‐SDF‐1α obviously enhanced the expression of COL1 compared to the other groups (**Figure** **9**A). Consistent with the in vitro tube formation assay, the HG‐AA_1:1_‐SDF‐1α group also significantly promoted the expression of CD31, exhibiting excellent in vivo angiogenesis (Figure 9B). To demonstrate the role of the composite hydrogel in activating the NO‐cGMP signaling pathway in vivo, immunofluorescence staining of PKG and p‐ERK was carried out. HG‐AA_1:1_‐SDF‐1α group showed enhanced expression of PKG and p‐ERK (Figure 9C,D). These results suggest that the composite hydrogel prepared herein could activate the NO‐cGMP pathway via sustained release of Arg‐CDs and Ca^2+^, and ultimately promote the formation of blood vessels and new bone. In summary, through the osteogenic–angiogenic coupling effect, the composite hydrogel exhibits a strong and effective bone regeneration bridging effect, and holds promise for the patients with impaired vascular and bone regeneration capacity.

## 3 Discussion

Recently, the application of MSCs to enhance bone tissue regeneration has become a research hotspot.^[^ ^18^ ^]^ To overcome the shortcomings of stem cell transplantation, the engineered HG‐AA_1:1_‐SDF‐1α could rapidly respond to the acidic microenvironment and release SDF‐1α, thereby recruiting endogenous BMSCs. The recruited BMSCs then promoted the metabolism of Arg‐CDs within composite hydrogel to produce abundant NO, which accelerated angiogenesis and provided an osteogenic microenvironment for bone regeneration.

Following bone injury, the local environment undergoes reduced blood supply and increased anaerobic metabolism. Moreover, with the CO_2_ exudation from the dead or dying cells, the production of lactic acid, and conversion of blood sugars, thus resulting in the formation of an acidic milieu, which can have detrimental effects on bone healing processes.^[^ ^19^ ^]^ The acidic microenvironment can, in particular, inhibit ALP expression and upregulate the expression of Gla protein (a known inhibitor of mineralization), thereby impeding osteogenic differentiation of BMSCs.^[^ ^20^ ^]^ Therefore, for improving the acidic microenvironment for optimal cell survival and proliferation, an HG‐AA_1:1_‐SDF‐1α composite hydrogel incorporating a Schiff base bond was developed herein, which is sensitive to acidic conditions.^[^ ^21^ ^]^ As 3D scaffolds, hydrogels have been applicated for bone remodeling due to their versatility, high biocompatibility and the ability to modify their composition.^[^ ^22^ ^]^ The ideal hydrogels must meet specific standards when used as an implanted scaffold for bone regeneration, such as no cytotoxic and no immunogenic response, excellent osteoinductive activity, analogous natural extracellular matrix (ECM) and biodegradable property.^[^ ^23^ ^]^ Compared to other biomaterials (e.g., zein, chitosan, alginate, poly(lactic‐co‐glycolic acid) et al.), GelMA hydrogel contains essential bioactive sequences such as arginine‐glycine‐aspartic acid (RGD) and matrix metalloproteinase (MMP) target sequences, thus is emerging as a promising biomaterial for supporting cell attachment, proliferation and bone regeneration.^[^ ^24^ ^]^ Besides, hyaluronic acid (HA) is a natural glycosaminoglycan which is found in human body and plays a critical role in tissue engineering own to its exceptional biocompatibility, biodegradability and non‐immunogenicity. Vicinal hydroxyl groups in HA can be oxidized to form aldehyde groups due to the periodate oxidation reaction.^[^ ^25^ ^]^ Therefore, the SDF‐1α can bind to HG‐AA_1:1_ via Schiff base bonds to obtain HG‐AA_1:1_‐SDF‐1α. After bone injury, HG‐AA_1:1_‐SDF‐1α intelligently released SDF‐1α in response to an acidic microenvironment. This controlled release mechanism was triggered by the rupture of the Schiff base bonds between SDF‐1α and HG‐AA_1:1_ under acidic conditions. Thus, HG‐AA_1:1_‐SDF‐1α plays a major role in maintaining cell activity and thus provides assurance for subsequent angiogenesis and osteogenic differentiation of HUVECs and BMSCs, respectively. This can be viewed as the first intelligent switch for the as‐engineered HG‐AA_1:1_‐SDF‐1α composite hydrogel.

After improvement in the bone injury microenvironment, the released SDF‐1α exhibited exceptional efficacy in recruiting endogenous BMSCs to bone injury sites, providing a substantial source of stem cells for bone repair.^[^ ^26^ ^]^ SDF‐1α is one of the most commonly used chemokines for recruiting MSCs.^[^ ^27^ ^]^ Besides, SDF‐1α recruits and promotes HUVECs proliferation and angiogenesis, which are vital processes in enhancing bone repair.^[^ ^28^ ^]^ The process involving utilization of endogenous stem cells via SDF‐1α is a superior alternative to conventional cell transplantation methods. Although cell transplantation has been previously considered to be a promising technique, it has also brought forward several significant limitations. These include the need for invasive surgical procedures for cell harvesting and implantation,^[^ ^29^ ^]^ potential risks of pathogen transmission‐induced severe infection during transplantation, substantial financial costs related to cell isolation and culture,^[^ ^3^ ^]^ and various risks associated with long‐term cell culture, such as genetic instability or contamination.^[^ ^30^ ^]^ By contrast, the novel HG‐AA_1:1_‐SDF‐1α composite hydrogel developed in this study facilitated the efficient migration of endogenous MSCs to bone injured sites. It addresses many of the limitations associated with cell transplantation techniques while still providing the necessary cellular resources for bone regeneration. Furthermore, the controlled release of SDF‐1α creates a chemotactic gradient that easily directs stem cells to the injured site. Importantly, this approach not only enhances the efficiency of MSCs recruitment, but also minimizes potential side effects that can possibly occur with systemic administration of growth factors or chemokines.

To promote the osteogenic differentiation of recruited BMSCs, the as‐engineered composite hydrogel led to the sustained release of Arg‐CDs and Ca^2+^, providing an excellent osteogenic microenvironment for osteogenesis. In this study, the released Ca^2+^ promoted the osteogenic differentiation of BMSCs. Notably, Ca^2+^ is also an important regulator of NOS activity. An increase in Ca^2+^ concentration can activate the calmodulin‐binding domain of eNOS, which leads to significant increase in eNOS activity. Therefore, recruited BMSCs could rapidly decompose Arg‐CDs to in situ produce NO at high Ca^2+^ concentrations, thus activating the NO‐cGMP signaling pathway and promoting angiogenesis for osteogenesis.^[^ ^31^ ^]^ This is the second intelligent switch for the HG‐AA_1:1_‐SDF‐1α composite hydrogel. Various studies have demonstrated an angiogenic effect of NO in bone repair. For instance, Lee et al. developed nanoparticles containing bioactive agents of zinc oxide that could obviously generate and release NO for angiogenesis.^[^ ^32^ ^]^ Cheng et al. constructed a photothermally‐induced S‐nitrosothiol (a class of photosensitive NO donors) delivery system to supply NO, which effectively stimulated angiogenesis and osteogenesis.^[^ ^33^ ^]^ Instead of relying on external NO donors, the HG‐AA_1:1_‐SDF‐1α composite hydrogel could utilize cellular metabolism to generate NO. Specifically, Arg‐CDs serve as precursors that are metabolized by the cells in the presence of Ca^2+^ to produce NO. The continuous release of Ca^2+^ from the composite hydrogel, coupled with the natural Ca‐rich environment of bone tissue, creates ideal conditions for sustained NO production. Consequently, the long‐term availability of Ca^2+^ and NO is crucial for maintaining angiogenic and osteogenic processes over an extended period. This approach ensures a more physiological and controlled release of NO, which mimics the natural processes in the body. Moreover, according to our previous study, Arg‐CDs showed better biological function in both antibacterial and osteogenic activities compared to L‐Arg.^[^ ^34^ ^]^ Therefore, we were confident that the constructed Arg‐CDs possessed a remarkable ability to generate more NO. Taken together, under the synergistic effect of Ca^2+^ and NO, the engineered HG‐AA_1:1_‐SDF‐1α composite hydrogel could efficiently achieve “coupling osteogenesis and angiogenesis”. The mechanism of “coupled osteogenesis and angiogenesis” suggests that neovascularization stimulates BMSCs through angiogenic paracrine signaling, thereby promoting cell proliferation or osteogenic differentiation. Such a close spatial and temporal coupling of angiogenic and osteogenic processes makes the HG‐AA_1:1_‐SDF‐1α composite hydrogel uniquely advantageous for bone repair.

## 4 Conclusion

In this study, HG‐AA_1:1_‐SDF‐1α composite hydrogel was successfully engineered to recruit endogenous BMSCs and realize “coupling osteogenesis and angiogenesis” for bone repair. HG‐AA_1:1_‐SDF‐1α intelligently released SDF‐1α for 35 days by recognizing the acidic microenvironment after bone injury, thus improving the acidic microenvironment and efficiently recruiting abundant endogenous BMSCs to the injured area. Further, the recruited BMSCs rapidly catalyzed metabolism of Arg‐CDs to produce NO in the presence of Ca^2+^, thus activating the NO/cGMP signaling pathway and promoting angiogenesis. Moreover, the release of Ca^2+^ from HG‐AA_1:1_‐SDF‐1α combined with the formation of blood vessels synergistically promoted bone repair after 8 weeks. Therefore, the engineered HG‐AA_1:1_‐SDF‐1α composite hydrogel showed enhanced “coupling osteogenesis and angiogenesis” and significantly accelerated bone regeneration inspired, which provides a new strategy for bone tissue engineering. Besides the excellent “coupling osteogenesis and angiogenesis” of HG‐AA_1:1_‐SDF‐1α for bone regeneration, the repair of weight‐bearing bone injuries is hard to accomplish own to its insufficient mechanical property. But we believe that our developed concept of “coupling osteogenesis and angiogenesis” is suitable for a variety of biomaterial systems, which provides the possibility to promote the repair of weight‐bearing bone injuries.

## 5 Experimental Section

### Preparation of Composite Hydrogels

The Arg‐CDs were obtained according to a previously method.^[^ ^34^ ^]^ Arg (500 mg) was heated at 240 °C for 3 h. Then the residue was dissolved in ultrapure water (20 mL). Next, the Arg‐CDs solution was centrifuged at 15,000 rpm. The supernatant was dialyzed against deionized water in a dialysis membrane (molecular weight cutoff = 1000 Da). Arg‐CDs were obtained after lyophilization.

To fabricate HA‐CHO, HA (1.5 g) was dissolved in deionized water (150 mL), and then sodium periodate (802 mg) was added and stirred for 2 h. The reaction was stopped by ethylene glycol (200 µL) and dialyzed against deionized water. The HA‐CHO was obtained after freeze‐dried.

To synthesize AA, SA (0.75 g) was dissolved in MES buffer (100 mL) under nitrogen protection and stirred at room temperature until its complete dissolution. Then, EDC (1.5 g) and NHS (2.25 g) were added to the SA solution and the contents were stirred for 15 min. Further, Arg‐CDs (10 mg) were dissolved in MES buffer (10 mL), added to the SA reaction mixture, and stirred overnight at room temperature. After dialysis with deionized water, AA was obtained via freeze‐drying.

To obtain HG‐AA‐SDF‐1α, G sponge (1 g), photo‐initiator (50 mg), and HA‐CHO (100 mg) were dissolved in deionized water (10 mL) at 37 °C. Next, AA (200 mg) was dissolved in deionized water (10 mL). The abovementioned solutions were mixed proportionally, and SDF‐1α was added and stirred well. HG‐AA‐SDF‐1α composite hydrogels were obtained after photo‐crosslinking (Wavelength: 405 nm; Time: 1 min) and Ca^2+^ coupling (CaCl_2_: 2% w/v).

### Physicochemical Characterization of Composite Hydrogels

The morphology of the Arg‐CDs was characterized by transmission electron microscopy (TEM, TECNAI G2 F20, FEI, USA). The XRD and UV–visible (UV–vis) absorption spectra were also performed via X‐ray diffractometer (Rigaku Ultima IV, Japan) and ultraviolet‐visible spectrophotometer (Thermo Fisher Scientific, USA). The molecular structures of HA‐CHO, AA, and HG‐AA‐SDF‐1α composite hydrogels were determined using a FTIR spectrometer (Nicolet 6700, Thermo Fisher Scientific, USA) in the wavelength range of 400–4000 cm^−1^ with a resolution of 2 cm^−1^.

After freeze‐drying, the morphology of the composite hydrogel was examined by scanning electron microscopy (SEM, FESEM, Zeiss, Germany). The porosities of the composite hydrogels were measured by the absolute ethanol displacement technique. The surface hydrophilicity of each sample was evaluated by a contact angle goniometer (K100, Kruss, Germany).

The release profiles of Arg‐CDs, Ca^2+^, and SDF‐1α from the composite hydrogels were measured by immersing the composite hydrogels in Ca^2+^/Mg^2+^‐free phosphate‐buffered saline (PBS, 2 mL) at pH 7.4 and 5.5 at 37 °C. The total concentration of the Arg‐CDs was determined by UV–vis (UV–vis) absorption spectroscopy. The Ca^2+^ release was measured by a colorimetric calcium assay kit. The release of SDF‐1α was detected using an SDF‐1α ELISA kit.

Young's modulus and mechanical properties of hydrogels was measured with a Piuma Nanoindenter (Optics 11) and universal testing machine (Hengyi Precision Instrument, China). The swelling parameters of the composite hydrogels were determined after immersing them in deionized water at 37 °C. The initial weight of the composite hydrogel was recorded as W_0_. The weight of the composite hydrogel at each time point was recorded as Wt. The change in mass was calculated by using the following formula: Change in mass (%) = (Wt−W_0_)/W_0_ × 100. Rheological properties of composite hydrogels were evaluated via rheometer (Anton Paar MCR92, China).

### Performance of Cell Adhesion and Proliferation of Composite Hydrogels

Briefly, 1 × 10^4^ cells were seeded in the lower chamber of a 24‐well transwell plate (Corning, #3415, USA) with a pore size of 3 µm. The composite hydrogels (HG, HG‐AA_4:1_, HG‐AA_2:1_, HG‐AA_1:1_) were placed into the upper chamber. Next, CCK‐8 assays were performed according to the manufacturer's instructions.

BMSCs and HUVECs were seeded on composite hydrogels. After 48 h of culture, cell viability was measured via live/dead assay (Thermo Fisher Scientific, MA, USA). Cell adhesion on the composite hydrogels was examined after staining with rhodamine phalloidin and 4′,6‐diamidino‐2‐phenylindole (DAPI). The morphology of the cells on composite hydrogels was characterized by SEM.

### Chemotaxis Assay of Composite Hydrogels

The effect of the composite hydrogel on the migration of BMSCs was determined by performing a scratch assay. Briefly, BMSCs were cultured in 6‐well plates (Corning, New York, USA) to allow the cells to completely cover the bottom of the plate. Further, scratches were made on the bottom of the plate with a 1‐mL pipette tip, then serum‐free α‐minimum essential medium (α‐MEM) was added as a culture medium to each well, and composite hydrogels were placed in the upper chambers of the corresponding wells. After co‐culturing for 24 h, healing of the scratch area was observed under an optical microscope (LSCM, LSM800, Zeiss, Germany).

Composite hydrogels were placed in the lower chamber of the transwell plate, and serum‐free α‐MEM culture medium (600 µL) was added. Next, BMSCs were seeded in the upper chamber of the transwell plate. After 24 h, crystal violet staining was performed to observe the migration of BMSCs. Finally, three randomly selected visual fields and the corresponding images were obtained and analyzed using an optical microscope (LSCM, LSM800, Zeiss, Germany).

### Angiogenic Potential Assessment of Composite Hydrogels

For measuring the tube formation ability of HUVECs induced by the composite hydrogels, Matrigel (BD BioCoat Matrigel, USA) was coated on the bottom of a 24‐well plate. HUVECs were seeded on the surface of Matrigel and cultured with extract liquid of composite hydrogels. After 3 h and 6 h of incubation, tube formation was observed under an optical microscope (LSM800, Zeiss, Germany). Further quantitative analysis was conducted by using ImageJ software.

The expressions for angiogenesis‐related genes (*VEGFA* and *CD31*) and protein (CD31) were respectively determined by qRT‐PCR and western blotting after 7 days, and the primer sequences were listed in Table S1 (Supporting Information). All the primers were synthesized by Invitrogen.

### Characterizations of in Vitro Osteogenesis of BMSCs

The composite hydrogels were placed in the upper chamber of 6‐well transwell plates (pore size: 8 µm, Corning, USA), and 1 × 10^5^ cells were cultured in the lower chamber. After 24 h, the osteogenic induction medium was added.

After 7 days of culture, the cells were fixed with 4% paraformaldehyde, and ALP staining was performed according to the manufacturer's instructions (Beyotime, China). The ALP activity was measured by ALP quantification kit (Beyotime, China). Alizarin red staining (Cyagen, Guangzhou, China) was performed to observe calcium deposits produced by BMSCs cultured with composite hydrogels after 21 days of osteogenic induction. Then, the calcium nodules were dissolved in perchloric acid and quantified using a microplate reader at a wavelength of 420 nm.

The expressions of osteogenesis‐related genes (*Alpl*, *Runx2*, *Col1a1*, and *Bglap*) and proteins (RUNX2, OPN, COL1, and OCN) were determined by qRT‐PCR and western blotting after 7 days of culture, and the primer sequences were listed in Table S2 (Supporting Information). All the primers were synthesized by Invitrogen.

### Exploration of Osteogenic Mechanism of Composite Hydrogels

The production of NO by BMSCs or HUVECs cultured with composite hydrogels was determined by the Griess method. Briefly, BMSCs or HUVECs were cultured with composite hydrogels for 3 days, and the nitrite concentration in the supernatant was quantified via the Total NO Assay (Beyotime, China). Moreover, cGMP levels were assessed using a cGMP ELISA kit (Bioboss, China). For systematic evaluation of the expression of associated signaling molecules in the NO‐cGMP pathway, proteins including PKG and ERK (Abcam, USA), were determined by western blotting.

### In Vivo Bone Repair Evaluation of Composite Hydrogels

Male Sprague–Dawley rats (weight: 300–350 g) were used to establish a critical‐sized skull defect model. All procedures followed the NIH Guide for the Care and Use of Laboratory Animals and were approved by the Institutional Animal Care and Use Committee of Soochow University (ECSU‐201700041). Briefly, rats were anesthetized with pentobarbital sodium solution (Sigma‐Aldrich). Next, the rat skin was disinfected and a longitudinal incision was made on the skull. Subsequently, critical‐sized defects (diameter: 5 mm) were created, and the composite hydrogels were implanted. After 4 and 8 weeks, respectively, skulls were harvested and fixed with 10% formalin. Micro‐CT (SkyScan, Belgium) was used to analyze the regenerated bone tissue in the defects. Tissue sections were stained with H&E, Masson's trichrome, CD31, osteocalcin (OCN), and Collagen‐1 (COL1) immunohistochemical staining to observe newly‐formed blood vessels and bone tissue. Immunofluorescence of CD105 was performed to observe the recruitment of BMSCs into the bone injured sites. PKG and p‐ERK immunohistochemical staining (Abcam, UK) was used to confirm in vivo activation of the NO‐cGMP signaling pathway.

### Statistical Analysis

Statistical analysis was performed using GraphPad Prism 8.0 software (USA). Data were presented as means ± SD. Details of biological replicates and statistical analysis were described in the corresponding Figure legends. One‐way or two‐way analysis of variance (ANOVA) coupled with Tukey's multiple comparison were used to evaluate differences between the groups. Unpaired two‐tailed t‐test was used to compare two groups. A probability value (p) less than 0.05 was considered statistically significant.

## Conflict of Interest

The authors declare no conflict of interest.

## Supporting information



Supporting Information

## Data Availability

The data that support the findings of this study are available in the supplementary material of this article.

## References

[1] a) A. I. Caplan , J. Cell. Physiol. 2007, 213, 341; 17620285 10.1002/jcp.21200

[2] a) P. W. Andrews , Biomater. Translat. 2021, 2, 294; 10.12336/biomatertransl.2021.04.004 PMC9255800 35837419

[3] Y. Wang , Z.‐b. Han , Y.‐p. Song , Z. C. Han , Stem Cells Int. 2012, 2012, 652034. 22685475 10.1155/2012/652034 PMC3363282

[4] A. K. Gaharwar , I. Singh , A. Khademhosseini , Nat. Rev. Mater. 2020, 5, 686.

[5] a) W. Zhang , N. Wang , M. Yang , T. Sun , J. Zhang , Y. Zhao , N. Huo , Z. Li , J. Orthopaedic Translat. 2022, 33, 41; 10.1016/j.jot.2022.01.002 PMC8858911 35228996

[6] K. Xi , Y. Gu , J. Tang , H. Chen , Y. Xu , L. Wu , F. Cai , L. Deng , H. Yang , Q. Shi , Nat. Commun. 2020, 11, 4504. 32908131 10.1038/s41467-020-18265-3 PMC7481196

[7] a) A. Massa , F. Perut , T. Chano , A. Woloszyk , T. A. Mitsiadis , S. Avnet , N. Baldini , Eur. Cells Mater. 2017, 33, 252; 10.22203/eCM.v033a19 28368079

[8] S. A. Yeh , J. Hou , J. Wu , S. Yu , Y. Zhang , K. Belfield , F. Camargo , C. Lin , Nat. Commun. 2022, 13, 393. 35046411 10.1038/s41467-022-27973-x PMC8770570

[9] Q. Chen , P. Shou , C. Zheng , M. Jiang , G. Cao , Q. Yang , J. Cao , N. Xie , T. Velletri , X. Zhang , Cell Death Differ. 2016, 23, 1128. 26868907 10.1038/cdd.2015.168 PMC4946886

[10] a) P. Kolar , T. Gaber , C. Perka , G. N. Duda , F. Buttgereit , Clin. Orthop. Relat. Res. 2011, 469, 3118; 21409457 10.1007/s11999-011-1865-3 PMC3183184

[11] Q. Qin , S. Lee , N. Patel , K. Walden , M. Gomez‐Salazar , B. Levi , A. W. James , Exp. Mol. Med. 2022, 54, 1844. 36446849 10.1038/s12276-022-00899-6 PMC9722927

[12] a) M. Marenzana , T. R. Arnett , Bone Res. 2013, 1, 203; 26273504 10.4248/BR201303001 PMC4472103

[13] J. O. Lundberg , M. T. Gladwin , E. Weitzberg , Nat. Rev. Drug Discovery 2015, 14, 623. 26265312 10.1038/nrd4623

[14] a) Y. Yang , T. Xu , Q. Zhang , Y. Piao , H. P. Bei , X. Zhao , Small 2021, 17, 2006598; 10.1002/smll.202006598 33705605

[15] A. D. Diwan , M. X. Wang , D. Jang , W. Zhu , G. A. Murrell , J. Bone Miner. Res. 2000, 15, 342. 10703937 10.1359/jbmr.2000.15.2.342

[16] a) U. Forstermann , T. Münzel , Circulation 2006, 113, 1708; 16585403 10.1161/CIRCULATIONAHA.105.602532

[17] M. Chen , Y. Li , X. Huang , Y. Gu , S. Li , P. Yin , L. Zhang , P. Tang , Bone Res. 2021, 9, 21. 33753717 10.1038/s41413-021-00138-0 PMC7985324

[18] a) J. Fu , Y. Wang , Y. Jiang , J. Du , J. Xu , Y. Liu , Stem Cell Res. Therapy 2021, 12, 1; 10.1186/s13287-021-02456-w PMC8254211 34215342

[19] a) B. Yu , C.‐Y. Wang , Trends Mol. Med. 2016, 22, 641; 27354328 10.1016/j.molmed.2016.06.002 PMC4969144

[20] X. Lin , Q. Wang , C. Gu , M. Li , K. Chen , P. Chen , Z. Tang , X. Liu , H. Pan , Z. Liu , J. Am. Chem. Soc. 2020, 142, 17543. 32960592 10.1021/jacs.0c07309

[21] a) C. Xue , L. Chen , N. Wang , H. Chen , W. Xu , Z. Xi , Q. Sun , R. Kang , L. Xie , X. Liu , Biomater. Translat. 2024, 5, 257,; 10.12336/biomatertransl.2024.03.004 PMC11681187 39734705

[22] N. Mamidi , F. F. De Silva , A. B. Vacas , J. A. Gutierrez Gomez , N. Y. Montes Goo , D. R. Mendoza , R. L. Reis , S. C. Kundu , Adv. Healthcare Mater. 2024, 27, 2401195. 10.1002/adhm.202401195 38824416

[23] K. Guillén‐Carvajal , B. Valdez‐Salas , E. Beltrán‐Partida , J. Salomón‐Carlos , N. Cheng , Polymers 2023, 15, 2762. 37447408 10.3390/polym15132762 PMC10346300

[24] N. Mamidi , F. Ijadi , M. H. Norahan , Biomacromolecules 2023, 25, 2075. 37406611 10.1021/acs.biomac.3c00279

[25] A. H. Pandit , N. Mazumdar , S. Ahmad , Int. J. Biol. Macromol. 2019, 137, 853. 31284008 10.1016/j.ijbiomac.2019.07.014

[26] X. Li , L. Wei , J. Li , J. Shao , B. Yi , C. Zhang , H. Liu , B. Ma , S. Ge , Appl. Mater. Today 2021, 22, 100942.

[27] Z. Jiang , L. Chen , L. Huang , S. Yu , J. Lin , M. Li , Y. Gao , L. Yang , Int. J. Nanomed. 2024, 19, 7751. 10.2147/IJN.S455469 PMC11297574 39099796

[28] L. Chen , C. Yu , Y. Xiong , K. Chen , P. Liu , A. C. Panayi , X. Xiao , Q. Feng , B. Mi , G. Liu , Bioactive Mater. 2023, 25, 460. 10.1016/j.bioactmat.2022.07.030 PMC10087917 37056272

[29] A. V. Cuomo , M. Virk , F. Petrigliano , E. F. Morgan , J. R. Lieberman , J. Bone Joint Surg. 2009, 91, 1073. 19411455 10.2106/JBJS.H.00303

[30] a) M. Honczarenko , Y. Le , M. Swierkowski , I. Ghiran , A. M. Glodek , L. E. Silberstein , Stem Cells 2006, 24, 1030; 16253981 10.1634/stemcells.2005-0319

[31] a) Y. Shen , H. Wang , H. Xie , J. Zhang , Q. Ma , S. Wang , P. Yuan , H. Xue , H. Hong , S. Fan , J. Orthopaedic Translat. 2024, 46, 53; 10.1016/j.jot.2024.03.003 PMC11131000 38808262

[32] J. K. Lee , D. S. Kim , S. Y. Park , S. W. Baek , J. W. Jung , T. H. Kim , D. K. Han , Adv. Sci. 2023, 10, 2205336. 10.1002/advs.202205336 PMC9951336 36581472

[33] Y. Cheng , Y. Huo , Y. Yu , P. Duan , X. Dong , Z. Yu , Q. Cheng , H. Dai , Z. Pan , Mater. Today Bio. 2024, 28, 101180. 10.1016/j.mtbio.2024.101180 PMC11364911 39221216

[34] J. Li , J. Ma , H. Sun , M. Yu , H. Wang , Q. Meng , Z. Li , D. Liu , J. Bai , G. Liu , Sci. Adv. 2023, 9, eadf8645. 37235658 10.1126/sciadv.adf8645 PMC10219602

