# Supplementary material for: Acid‐Triggered Dual‐Functional Hydrogel Platform for Enhanced Bone Regeneration
Source: Adv Sci (Weinh). 2025 Jan 27;12(11):2415772. doi: 10.1002/advs.202415772 (PMC11923904; doi:10.1002/advs.202415772)
Supplement: Supplementary file 1 — Supporting Information [file ADVS-12-2415772-s001.docx]

**Supporting Information**

**Figures**


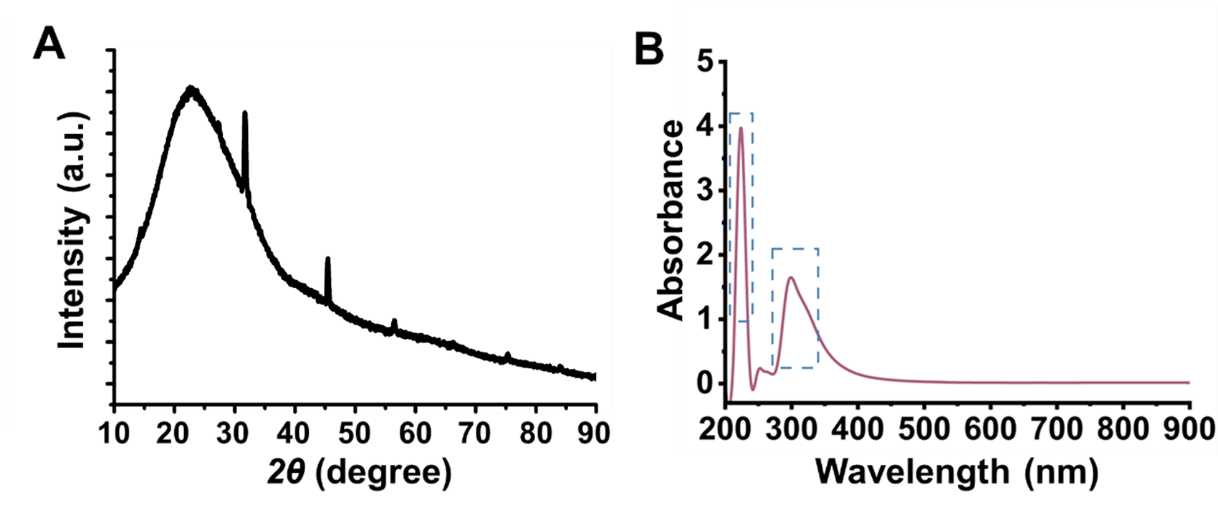


**Figure S1.** (A) XRD and (B) UV-visible (UV-vis) absorption spectra of Arg-CDs.


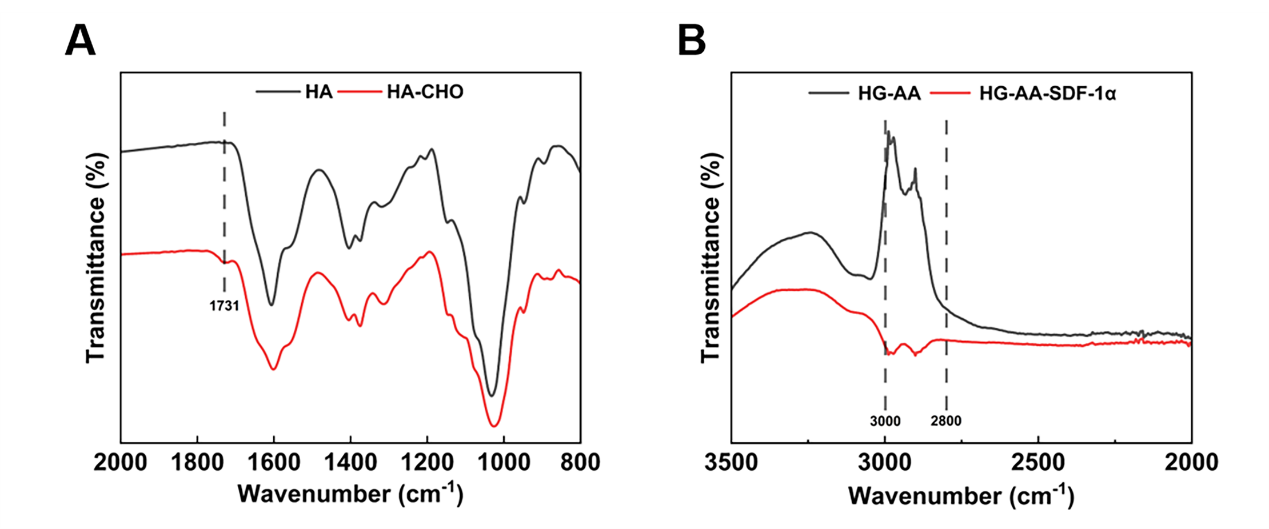


**Figure S2.** (A) FTIR spectra of HA, HA-CHO. (B) FTIR spectra of HG-AA and HG-AA-SDF-1α.


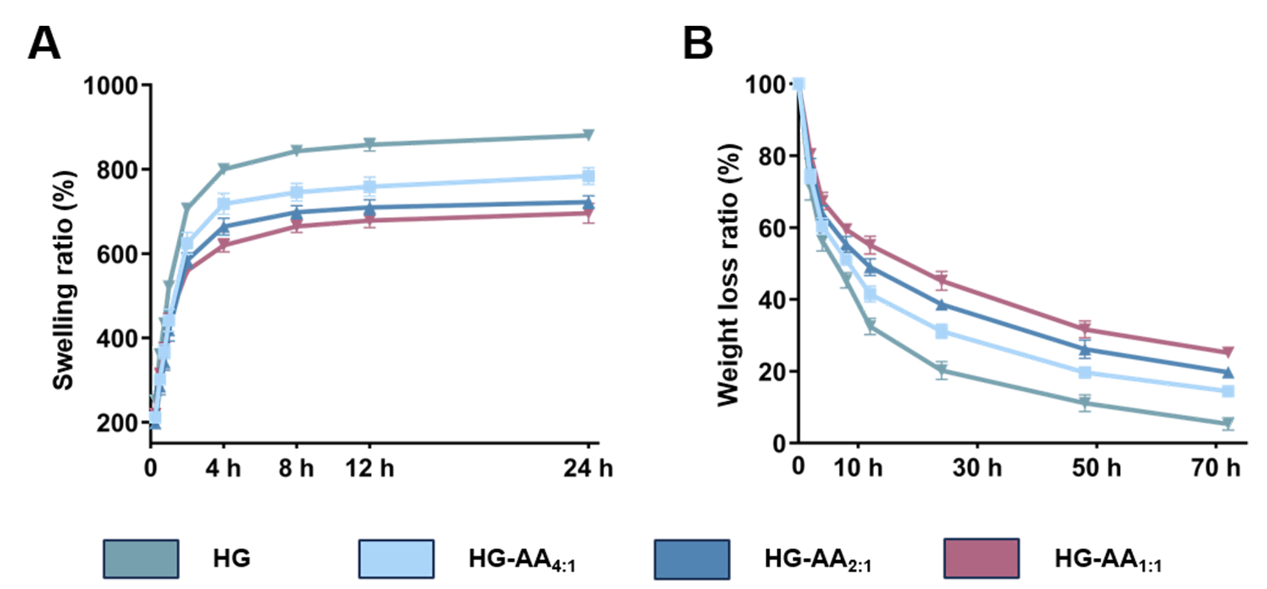


**Figure S3.** (A) Swelling ratio curves of composite hydrogels. (B) Weight loss ratios of composite hydrogels.


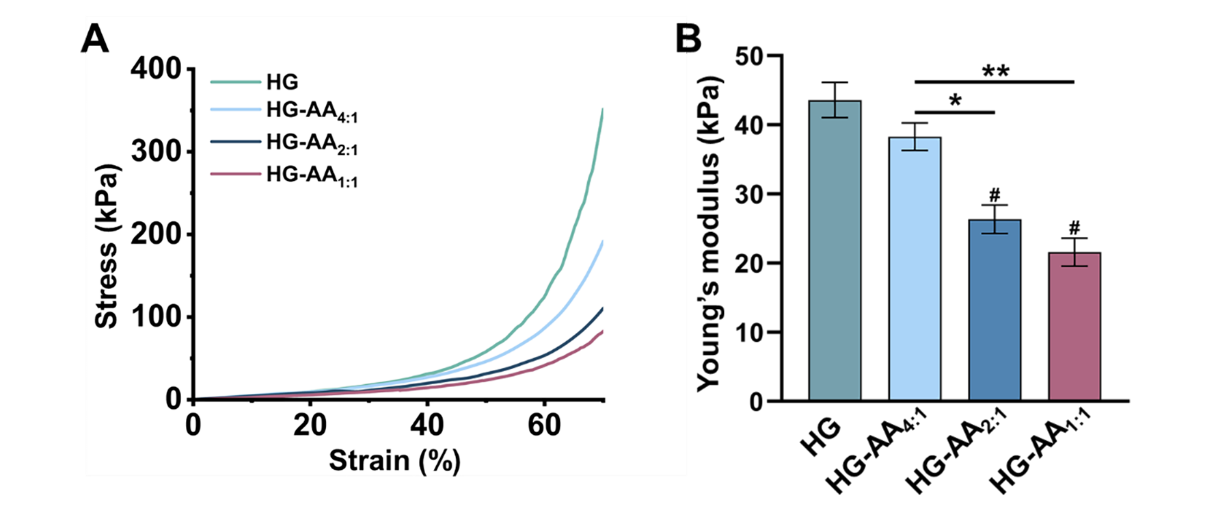


**Figure S4.** (A) Stress-strain curve and (B) Quantitative analysis of Young’s modulus of composite hydrogels. Data are expressed as the mean ± SD (n=3, ^#^ compared with HG group, ^*^ compared with each other; ^*^*p* < 0.05, ^**^*p* < 0.01)


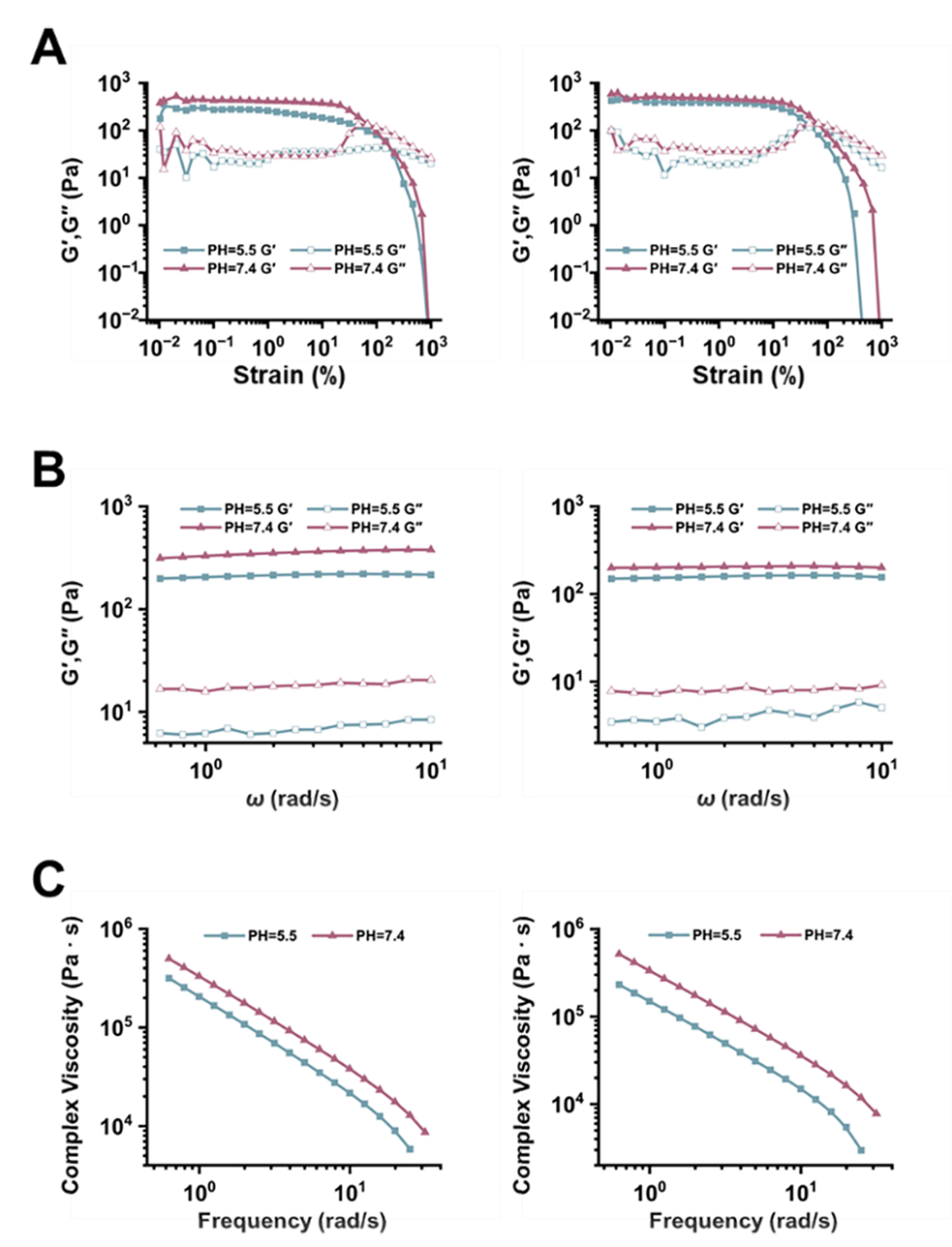


**Figure S5.** Rheological properties of composite hydrogels. (A) Strain sweep measurement of the composite hydrogels with a set frequency of 1 Hz. (B) Variations of storage modulus G′ and loss modulus G″ of the composite hydrogel as a function of angular frequency at 37°C and 25 °C. (C) The shear-thinning properties of the composite hydrogels at 37°C and 25 °C.


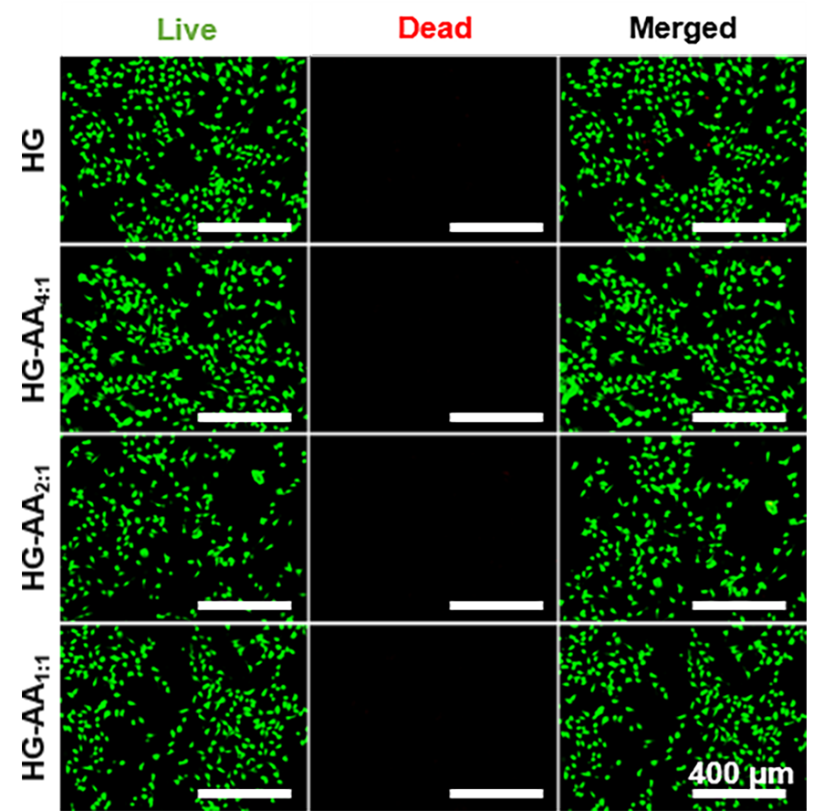


**Figure S6.** Representative Live/Dead staining images of HUVECs cultured on different composite hydrogels.


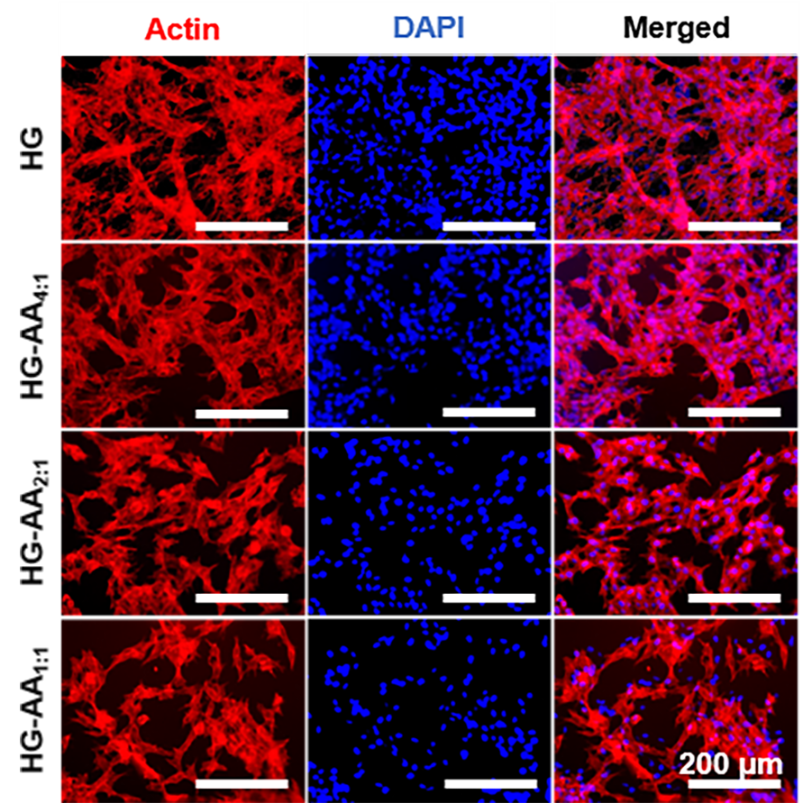


**Figure S7.** Representative cytoskeleton staining images of HUVECs on the composite hydrogels.


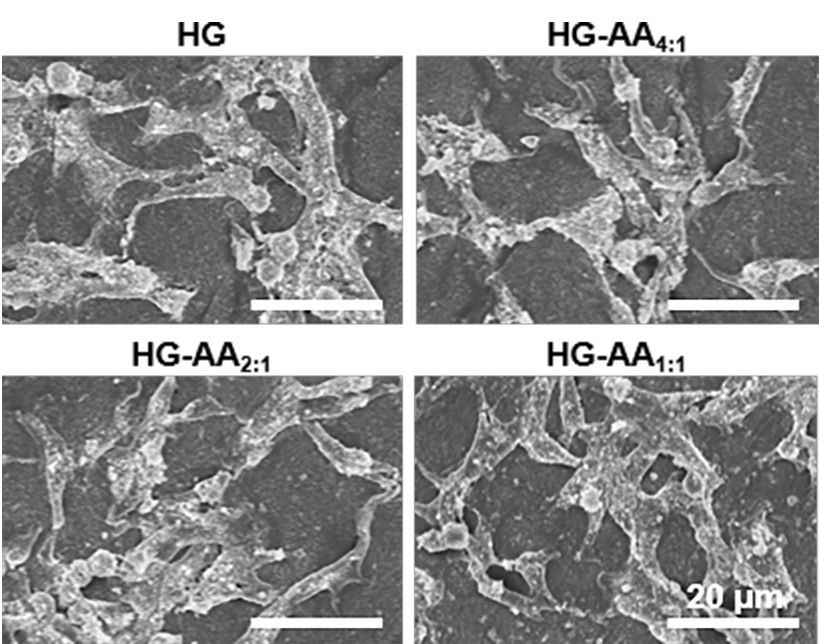


**Figure S8.** SEM images of cell morphology on the surface of composite hydrogels.


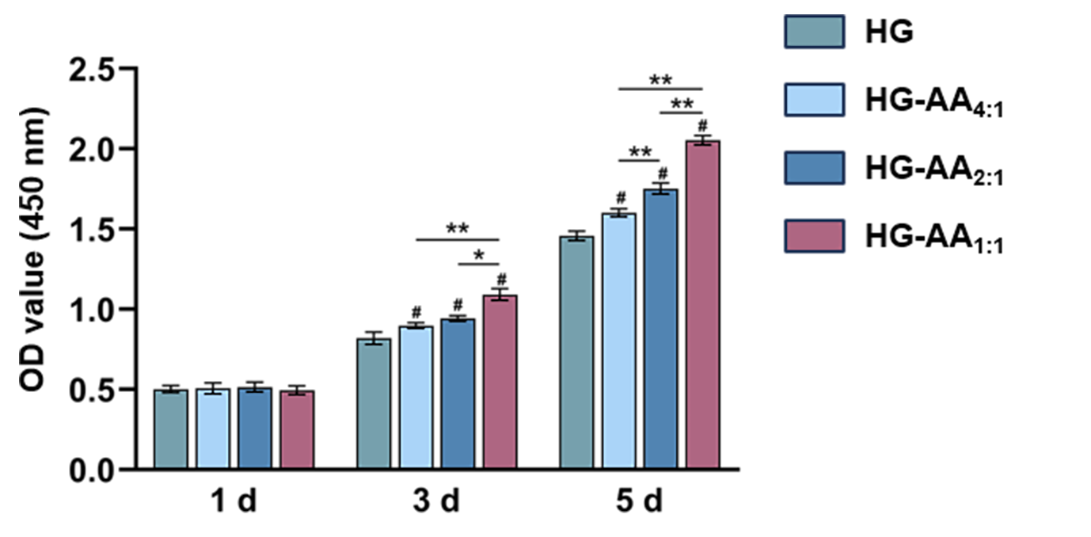


**Figure S9.** Quantitative analysis of cell proliferation assay (n = 8 images). Data are expressed as the mean ± SD (n=6, ^#^ compared with HG group, ^*^ compared with each other; ^*^*p* < 0.05)


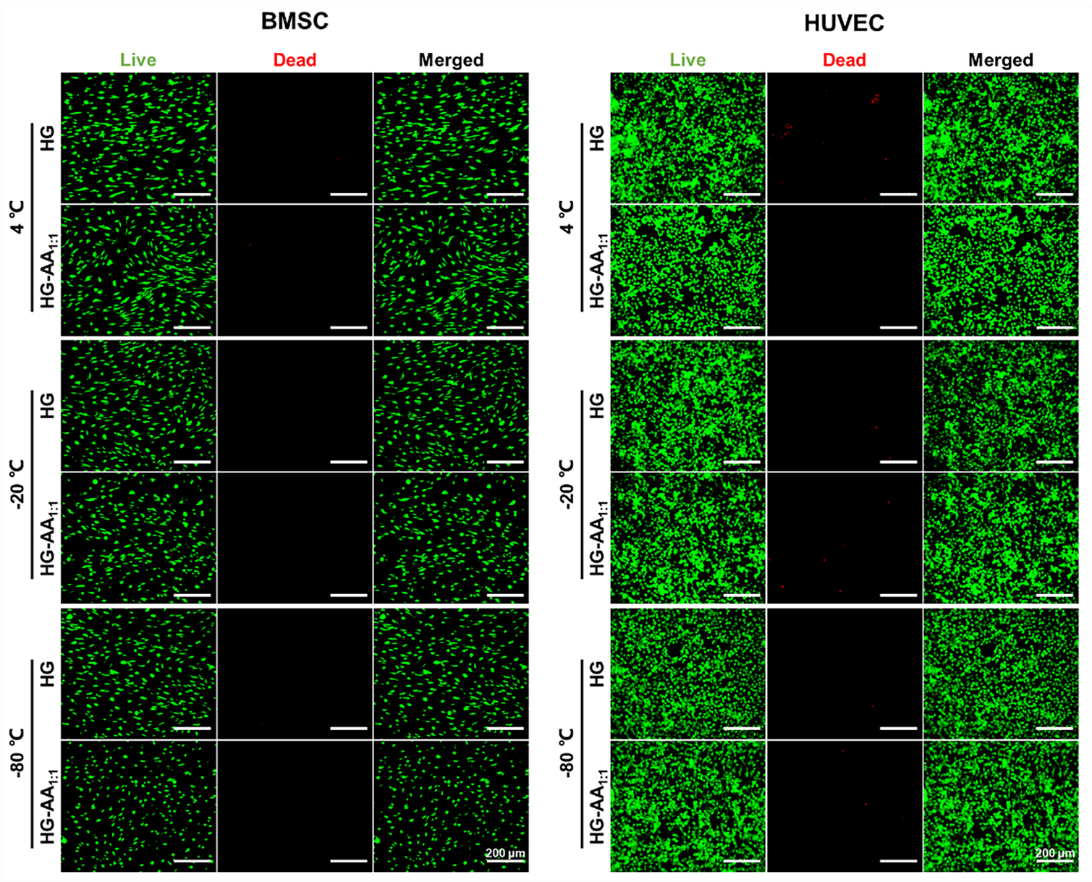


**Figure S10.** Representative Live/Dead staining images of BMSCs and HUVECs cultured on different composite hydrogels.


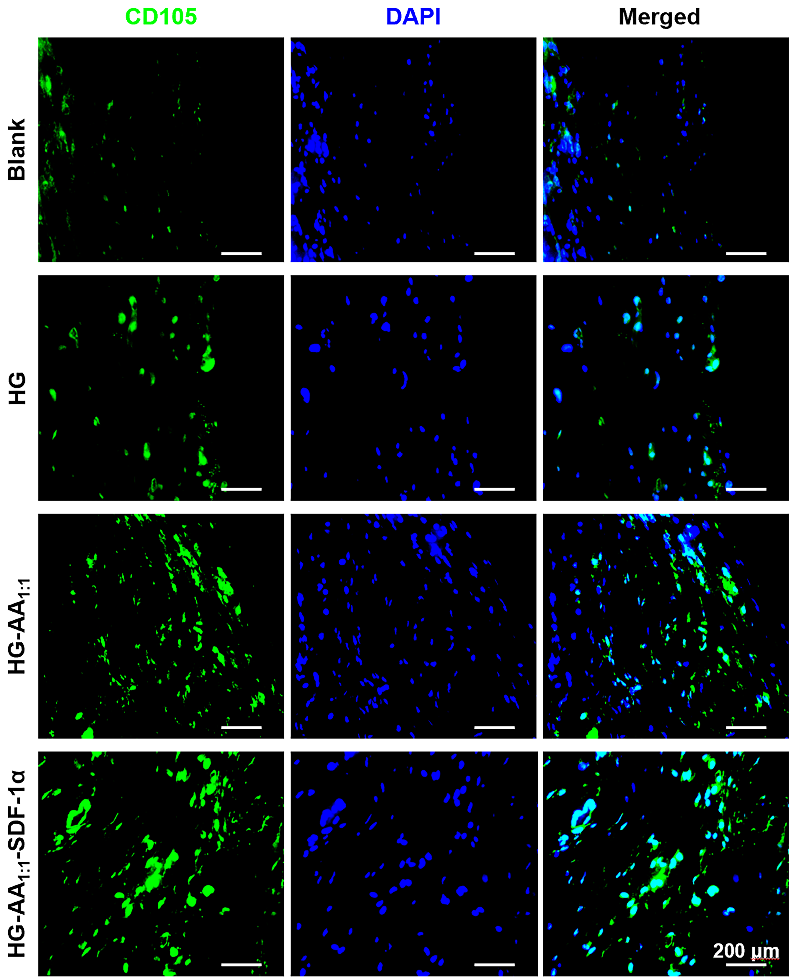


**Figure S11.** Immunofluorescence of CD105.

**Table 1**. Primer sequences used for RT-qPCR.

| Gene | Primer sequences (5'-3') |
| --- | --- |
| *CD31* | Forward 5'-ACCCTGGAGTGCCTTGTGGAC-3' |
|  | Reverse 5'-AGACCCGAGCCTGAGGAATGAC-3' |
| *VEGF-A* | Forward 5'-CACGACAGAAGGAGAGCAGAAGTC-3' |
|  | Reverse 5'-GTCTCAATCGGACGGCAGTAGC-3' |
| *GAPDH* | Forward 5'-GTCTCCTCTGACTTCAACAGCG-3' |
|  | Reverse 5'-ACCACCCTGTTGCTGTAGCCAA-3' |

**Table 2.** Primer sequences used for RT-qPCR.

| Gene | Primer sequences (5'-3') |
| --- | --- |
| *Alpl* | Forward 5'-TATGTCTGGAACCGCACTGAAC-3' |
|  | Reverse 5'-CACTAGCAAGAAGAAGCCTTTGG-3' |
| *Bglap* | Forward 5'-CATGAAGGCTTTGTCAGACT-3' |
|  | Reverse 5'-CTCTCTCTGCTCACTCTGCT-3' |
| *Col1a1* | Forward 5'-CAGGCTGGTGTGATGGGATT-3' |
|  | Reverse 5'-CCAAGGTCTCCAGGAACACC-3' |
| *Runx2* | Forward 5'-TCTTCCCAAAGCCAGAGCG-3'  Reverse 5'-TGCCATTCGAGGTGGTCG-3' |
| *Actin* | Forward 5'-CTCATGCCATCCTGCGTCTG-3' |
|  | Reverse 5'-GGCAGTGGCCATCTCTTGCT-3' |
